# Internal Curing Effect of Pre-Soaked Zeolite Sand on the Performance of Alkali-Activated Slag

**DOI:** 10.3390/ma14040718

**Published:** 2021-02-03

**Authors:** Guang-Zhu Zhang, Han-Seung Lee, Xiao-Yong Wang, Yi Han

**Affiliations:** 1 Songsim Global Campus, Catholic College, The Catholic University of Korea, Bucheon-si 14662, Korea; zhangks@catholic.ac.kr; 2 Department of Architectural Engineering, Hanyang University, Ansan-si 15588, Korea; ercleehs@hanyang.ac.kr; 3 Department of Architectural Engineering, Kangwon National University, Chuncheon-si 24341, Korea; 4 Department of Integrated Energy and Infra System, Kangwon National University, Chuncheon-si 24341, Korea; hanyii@kangwon.ac.kr

**Keywords:** pre-soaked zeolite sand, alkali-activated slag, internal curing, autogenous shrinkage

## Abstract

This study clarifies the effects of pre-soaked zeolite sand as an internal curing material on the hydration, strength, autogenous shrinkage, and durability of alkali-activated slag (AAS) mortars. The liquid-to-binder ratio (L/b) of all of the AAS mortars was 0.55. Sodium hydroxide solution was used as an alkali activator and an internal curing liquid. Calcined zeolite and natural zeolite sand replaced the standard sand at 15% and 30%, respectively. The setting time, autogenous shrinkage, compressive strength, ultrasonic pulse velocity, and surface electrical resistivity were tested. The following conclusions were drawn: (1) The addition of zeolite significantly reduces the autogenous shrinkage of AAS mortar. Compared with the control group, 30% calcined zeolite reduced the autogenous shrinkage by 96.4%. Moreover, the autogenous shrinkage of the AAS mortars was noticed in two stages (a variable temperature stage and an ambient temperature stage), and the two stages split at one day of age. (2) The compressive strength of all of the specimens increased as the zeolite sand content increased, and the highest compressive strength was obtained for AAS combined with 30% natural zeolite sand. (3) Internal curing accelerated the formation of the second peak of heat flow and reduced the accumulated heat release. (4) Calcined zeolite sand delayed the setting time of the AAS mortars. (5) The addition of zeolite significantly reduced the surface electrical resistivity of the AAS mortars. In summary, zeolite sand is extremely useful as an internal curing agent to reduce autogenous shrinkage and to increase the compressive strength of AAS mortars.

## 1. Introduction

Although ordinary Portland cement (OPC) is a widely used inorganic binder in modern construction, researchers have considered the possibility of using alkali-activated slag (AAS) to replace some of the OPC in construction due to the negative impact of OPC on the environment [1]. Because less energy is needed in the AAS production process, carbon emissions can be reduced [2,3]. Alkali-activated slag concrete is prepared by blending blast furnace slag, an alkali-activated solution, a fine aggregate, and a coarse aggregate [4].

One of the main reasons restricting the use of AAS is that its autogenous shrinkage is significantly higher than that of OPC [5]. The cement composition and fineness of slag [6,7], the form and quantity of the alkali activator, and the curing conditions [8] are all factors that have been documented to impact cement shrinkage in the production of slag. Researchers have attempted to mitigate the autogenous shrinkage of the AAS system by employing different approaches to decrease the autogenous shrinkage of the OPC, considering the similarity of the autogenous shrinkage between the AAS and OPC systems.

In concrete structures, shrinkage is the primary cause of related cracking and volume instability. Autogenous shrinkage is caused by the self-desiccation of concrete, leading to higher internal stresses and early cracking [9]. Autogenous shrinkage is closely related to cement particle hydration in OPC systems [10]. At present, internal curing is considered to be one of the proven approaches for reducing the autogenous shrinkage of OPC systems [11,12]. Porous materials are often used as internal curing agents, which can be thought of as reservoirs that absorb much of the internal curing liquid and that are released when needed during the hydration reaction [13]. Based on this statement, more and more researchers are applying internal curing to AAS systems.

First, superabsorbent polymers (SAPs), as one of the most effective internal curing agents for reducing autogenous shrinkage, have been widely studied. Song et al. [14] reported that the autogenous shrinkage of AAS can be fully compensated when the amount of SAP exceeds 0.6%, but more voids can be created in the introduced SAPs, resulting in a decrease in compressive strength. Similar results were reported by Oh et al. [15], where the incorporation of SAPs effectively reduced autogenous shrinkage (approximately 25% of the control group), but AAS mortars with SAPs exhibited a loss of strength. Li et al. [16] measured the autogenous shrinkage strain of AAS with different amounts of SAPs up to seven days of age. It became clearer that autogenous shrinkage cannot be completely eliminated, no matter how high the SAP content in AAS. SAPs are an ideal material for reducing the autogenous shrinkage of AAS mortars, but it is not worthwhile to consider them for this purpose, as they cause a considerable loss of compressive strength.

Second, Bentz and Sakulich [17] found that lightweight aggregates (LWAs) as an internal curing agent can completely reduce autogenous shrinkage and fortunately, LWAs only minimally reduce compressive strength. Therefore, other researchers have also focused their attention on finding new LWA materials that can be used to reduce autogenous shrinkage without causing loss of strength. Darshan et al. [18] reported the use of expanded shale (absorption capacity of 18%) as an internal curing agent and a shrinkage-reducing admixture to mitigate the autogenous shrinkage of AAS mortars. It was found that at 28 days of age, the addition of 7.5% of the shrinkage-reducing admixture reduced the autogenous shrinkage by approximately 75%, while replacing 20% of the normal aggregates with expanded shale reduced the autogenous shrinkage by approximately 50%. Moreover, Darshan found that although the two methods worked by different mechanisms, the shrinkage-reducing admixture performed more effectively. Other researchers have focused on recycled aggregates to implement internal curing effects in AAS systems because of the high liquid absorption of recycled aggregates. Lee et al. [19] found no loss of compressive strength, and autogenous shrinkage was effectively reduced but not completely eliminated at an early age. This may be due to the large particle sizes of recycled aggregate, resulting in a smaller surface area to provide the internal curing liquid. Additionally, recycled aggregates have small internal pores, which results in a slow release of the internal curing liquid. Another reason for this is that the recycled aggregate material itself may partially react with the AAS system to generate products that block the pathway for the internal curing liquid to be released.

Finally, previous studies have reported that natural zeolite sand can be used as an internal curing agent for OPC systems due to its fine internal pore structure. Erfanimanesh and Sharbatdar [20] found the compressive strength of geopolymer mortar containing zeolite and slag was much higher than that of ordinary cement mortar. Zhang et al. [21] used pre-soaked zeolite as an internal curing agent to reduce the autogenous shrinkage of a high-strength engineered cementitious composite. At four weeks, autogenous shrinkage was reduced by 60% and showed similar levels of compressive strength to the control group. Other researchers have found that modification methods such as calcination can improve the internal curing effect of zeolite sand. For example, Wang et al. [22] studied zeolites calcined at 500 °C for 30 min for use as internal curing agents and found that the efficiency of said calcined zeolites in reducing autogenous shrinkage was significantly improved, and 30% of calcined zeolites reduced the shrinkage by 36% at 28 days. Similar findings were reported in the study by Zhang et al. [23], who found that calcination improved the internal pore structure of zeolite sand, enabling it to retain more water, thereby reducing the autogenous shrinkage of ultra-high-performance concrete. However, it should be noted that after calcination at 500 °C, the absorption ability of zeolite sand increased, hence the spalling resistance of concrete containing calcined zeolite sand decreases at elevated temperatures [24,25,26]. In addition, the calcination may damage the zeolite sand. In summary, calcination of zeolite sand raises the question of the useability of this material at elevated temperatures.

Based on the above review of the literature on internal curing, we found the weak points of previous studies to be as follows: (1) SAPs effectively reduce autogenous shrinkage but cause a significant loss of compressive strength; (2) the currently available lightweight aggregates are capable of reducing autogenous shrinkage without causing a loss of compressive strength, but their efficiency in reducing autogenous shrinkage is not satisfactory; (3) pre-soaked lightweight aggregates are mostly studied in OPC systems, while research on their use in AAS systems is very limited. Therefore, it is of great interest to find a new LWA as an internal curing material to alleviate the autogenous shrinkage of AAS mortars without causing a loss of strength.

In this study, the feasibility of zeolite sand as an internal curing agent for AAS mortars was experimentally investigated as follows: first, zeolite sand was investigated as a new internal curing agent to significantly eliminate autogenous shrinkage. Second, to obtain a more significant autogenous shrinkage effect, natural zeolite sand was modified by the calcined method, and the effect of the two different zeolite sands on the internal curing of AAS systems was evaluated. Finally, the internal curing mechanism of zeolite sand for AAS systems was explored to fill the gap in the literature regarding the use of zeolite sand as an internal curing agent for AAS systems.

## 2. Materials and Methods

### 2.1. Materials and Sample Preparation

Based on our previous study [22], calcination is an effective method for improving the internal pore structure of zeolite to increase its porosity. Therefore, in this study, we used the same pretreatment method to prepare modified zeolite. The detailed procedures are described in [22]. The fineness and density of the granulated ground blast furnace slag (GGBFS) were 405 m^2^/kg and 2.68 kg/m^3^, respectively. The chemical compositions of the slag and zeolite sand are shown in Table 1. In this study, the activator used to accelerate the slag reactivity was sodium hydroxide pellets (analytical grade >98%; Daejung, Siheung-Si, Korea), which had a purity of 97%. Before mixing with powders, the activator was dissolved in deionized water and allowed to cool to room temperature for 24 h.

**Figure 1:**
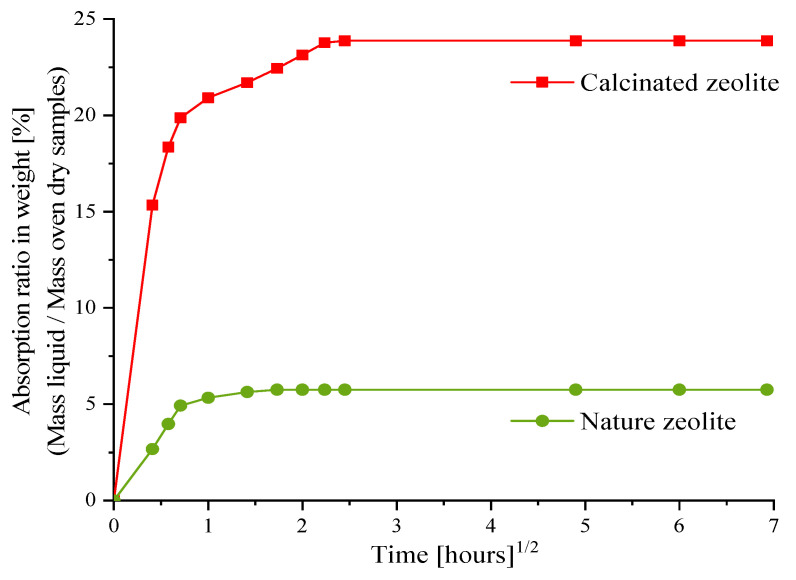
Square root of time vs. NaOH solution absorption of natural and calcined zeolite sand.

In this study, two types of shrinkage reduction agents (natural and calcined zeolite sand) were used. The particle size of the zeolite sand ranged from 1 to 3 mm. The activator solution’s absorption of the zeolite sand was measured by the tea-bag method [27]. Absorption was calculated as the ratio of the measured wet mass to the dry mass of the zeolite sand. The average values of two tea-bag tests were used. Figure 1 shows the development curves of the activator solution absorption of the zeolite sand. After 6 h, the zeolite sand was almost saturated and no longer absorbed the NaOH liquid. The absorption rates of the natural and calcined zeolite sand were 5.58 and 23.80 wt.%, respectively. This phenomenon was mainly a result of the calcined method improving the pore structure of the zeolite sand, and because the calcined zeolite is able to store a greater amount of alkali solution.

All of the mix proportions of the AAS mortars are shown in Table 2. The mass proportions of slag, standard sand, and liquid were in a ratio of 1, 2, and 0.55, respectively, for all AAS mortars. In addition, the mixtures based on unity volume shown in the right side of Table 2 can be determined based on the mass and density of individual components of AAS mortar. Labels denoting NZ and CZ represent the internal curing of the AAS mortars with natural and calcined zeolite sand, respectively. The labels denoting a number represent the zeolite mass fraction of standard sand. A control group of AAS mortars without internal curing was labeled as AAS-0. The liquid-to-binder ratio (L/b) of the AAS mortar without internal curing (AAS-0) was 0.55. For the specimens containing zeolite sands, the basic l/b (corresponding to the liquid not absorbed by the zeolite) of the AAS with internal curing was the same as that of AAS-0. The additional liquid (absorbed by the zeolite sand)-to-binder ratio was marked as L/b_IC_. The total L/b of the AAS mortars with internal curing was the sum of the basic L/b and L/b_IC_, and the NaOH solution absorption of the natural and calcined zeolite sand was 5.58 and 23.80 wt.% of the dry zeolite mass. Therefore, the total l/b of AAS-NZ15, AAS-NZ30, AAS-CZ15, and AAS-CZ30 was 0.567, 0.584, 0.621, and 0.692, respectively. Ideally, similar to OPC systems, the replacement ratio of zeolite sand should be decided based on the needed internal curing water and absorption/desorption capacity. With the AAS system, it is difficult to find the theoretical needed “internal curing liquid.” In this study, zeolite sand’s replacement ratios (15% and 30%) were taken from reference [23]. In future studies, we will test the chemical shrinkage of AAS according to ASTM C1608 [28], and the results may be used in mixture design to quantify the LWA replacement ratio.

The slag, standard sand, and pre-soaked zeolite sand were stirred using a mortar mixer at a low speed for 30 s. The NaOH liquid was added to all powders and stirred at a low speed for 2 min. Then, it was stopped for 30 s, and the under-agitated powder was scraped off of the mortar mixer’s inner wall. The mixing continued at a high-speed for another 120 s.

### 2.2. Test Methods

#### 2.2.1. Setting Time

The setting time of the AAS mortars was determined by the Vicat needle test, which was conducted in accordance with ASTM C191 [29]. All of the mortars were prepared at 20 ± 1 °C. During the test, the mortars were covered with a plastic film to avoid the evaporation of water from the mortar.

#### 2.2.2. Autogenous Shrinkage

The measurement of autogenous shrinkage was performed according to ASTM C1698 [30]. Since the internal relative humidity and internal temperature act as critical factors influencing the autogenous shrinkage of concrete [31], the autogenous shrinkage, internal relative humidity, and internal temperature of the AAS mortars were measured simultaneously in this study. We modified the ASTM C1698 measuring device by inserting a sensor in the center of the corrugated plastic tube to monitor the internal relative humidity and internal temperature. Moreover, the sensor was protected by a polyvinyl chloride tube. Details of the modified measuring device can be found in a previous study [32]. All mortars were prepared at 20 ± 1 °C and cured in a constant temperature chamber at 20 ± 1 °C.

#### 2.2.3. Compressive Strength Test

The purpose of this study was to investigate the effects of zeolite sand on the autogenous shrinkage, early-age compressive strength, hydration heat, and various other properties of AAS mortars. Therefore, the three- and seven-day compressive strength of the AAS mortars was tested according to ASTM C39 [33]. The size of the specimens for the compressive strength test was 50 × 50 × 50 cm^3^. The fresh mortars were cast into a mold and cured for one day. After this, the mortars were removed from the mold and sealed by polyethylene film until the test age. Three specimens were tested per group of experiments, and the average of the three data obtained was used to discuss the compressive strength.

#### 2.2.4. Isothermal Calorimetry

The hydration kinetics of all of the AAS mortars were measured using a TAM-Air (TA Instruments, New Castle, DE, USA). The adhesives were cast for 72 h at 20 °C. In previous studies, most researchers studied pastes for experimental subjects. However, in this study, we investigated the effect of pre-soaked zeolite sand (a fine aggregate) on the hydration kinetics in AAS systems. Therefore, mortar specimens were used for the isothermal calorimetric test. In order to mix the adhesives adequately, all mortars were mixed using a mixer, and 10 g of each mortar was transferred to separate glass bottles for testing.

#### 2.2.5. Ultrasonic Pulse Velocity (UPV) Test

The strength and uniformity of AAS can be reflected in a UPV test. UPV can be used for assessing the structural integrity of specimens and for determining where voids occur in said specimens. Days three and seven were used for the test ages to examine the impact of natural and calcined zeolite sand on the structural integrity of specimens in the early stages. A portable non-destructive digital indicating tester supported by Proceq Pundit Lab was used for the UPV test. All specimen tests were carried out under longitudinal P-wave velocity according to the ASTM C 597-16 standard [34]. UPV test results were obtained for the time it took the pulse to between the transmitting and receiving ends of the device, i.e., the time it took to pass through the specimen. The pulses were 54 kHz. The average of three UPV measurements was used.

#### 2.2.6. Surface Electrical Resistivity (SER)

The electrical resistivity was also measured using rectangular samples with a diameter of 102 mm and a length of 204 mm, using a four-point Wenner surface test system according to the surface resistivity test method of AASHTO T 358 [35]. Three specimens were tested at the ages of three and seven days for each mortar mixture.

## 3. Results and Discussion

### 3.1. Setting Time

**Figure 2:**
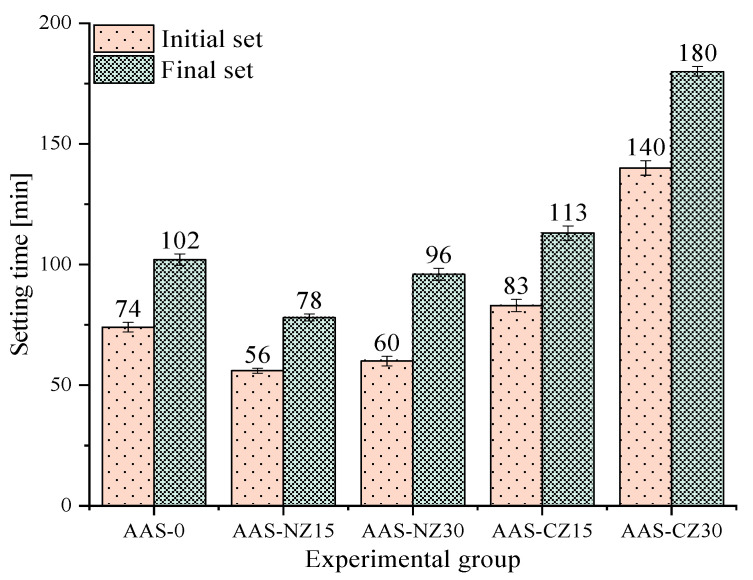
The setting times of all of the alkali-activated slag (AAS) mortars.

Figure 2 shows the initial and final setting times of all of the AAS mortars. The setting times of the AAS mortars with internal curing by natural zeolite sand (AAS-NZ15 and AAS-NZ30) were shorter than that of AAS-0. The addition of natural zeolite released the NaOH liquid, resulting in an increase in the ion concentration of the mortars with internal curing and in an acceleration of the slag reaction. On the contrary, compared to AAS-0, the setting times occurred later for the AAS mortars with internal curing by calcined zeolite sand (AAS-CZ15 and AAS-CZ30). All groups of specimens had the same basic L/b. The internal pore structure grew more when natural zeolite was calcined [22], and the addition of calcined zeolite inside the AAS mortars introduced additional pores to reduce the compressive strength, resulting in significantly prolonged Vicat needle test values. In summary, pre-soaked natural zeolite sand reduced the setting time, but pre-soaked calcined zeolite sand delayed the setting time. The reduction of setting time may be helpful for cold weather construction, and the delaying of setting time may be beneficial for hot weather construction. Moreover, the addition of calcined zeolite sand prolonged the setting time of the AAS mortars, making it possible to alleviate the problem of the AAS mortars being challenging to process in cast-in-place concrete due to rapid setting.

### 3.2. Autogenous Shrinkage, Internal Relative Humidity, and Internal Temperature

#### 3.2.1. Development of Autogenous Shrinkage

**Figure 3:**
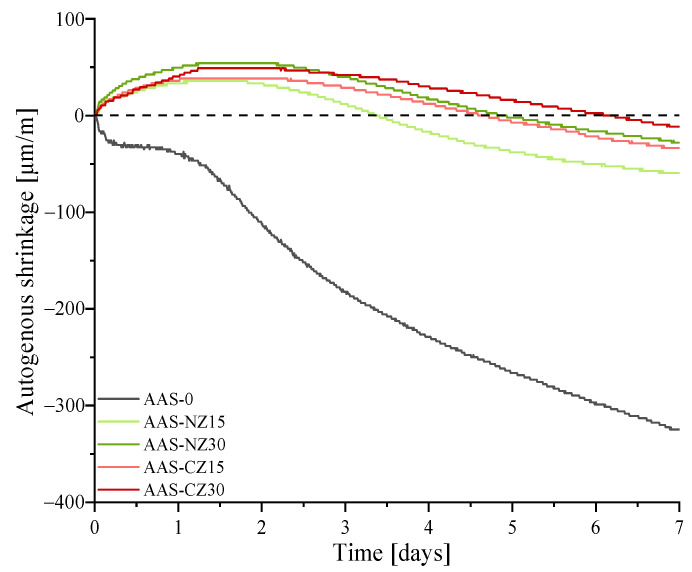
Autogenous shrinkage development curves of the alkali-activated slag (AAS) specimens from the final setting time to 7 days.

Figure 3 shows the autogenous shrinkage development curves of the control specimen (AAS-0), the AAS mortars blended with natural zeolite sand (AAS-NZ15 and AAS-NZ30), and the modified zeolite sand (AAS-CZ15 and AAS-CZ30).

The autogenous shrinkage of AAS-0 increased from zero (final setting time) to seven days. For the other four groups, all of the AAS mortars with internal curing first showed expansion and then showed shrinkage. A similar phenomenon was found in a past study by Chen. Chen et al. [36] found that the amount of internal curing liquid affected the expansion duration and that AAS mortars continued to expand when the amount of internal curing liquid was sufficient. The occurrence of expansion is mainly attributed to the production of expansive Si-rich gel [17]. Moreover, the expansion of AAS mortars with the same amount of zeolites resulted in a similar strain. Herein, the expansion strains of AAS-NZ15, AAS-NZ30, AAS-CZ15, and AAS-CZ30 were 35.76, 54.44, 38.03, and 49.77 μm/m, respectively.

At seven days of age, the autogenous shrinkage strain of AAS-0, AAS-NZ15, AAS-NZ30, AAS-CZ15, and AAS-CZ30 was −324.68, −59.51, −28.24, −33.52, and −11.65 μm/m, respectively. The autogenous shrinkage of all of the AAS mortars was almost significantly attenuated by adding internal curing agents to the AAS mortars. This behavior happened because the internal curing liquid was released to compensate for the loss of activation solution of the AAS mortars caused by rapid hydration. An internal curing liquid travels to wet the drying capillary pores of zeolite sand in the surrounding AAS mortars, leading to decreased capillary stress and decreased autogenous shrinkage [36].

Regarding the cost, due to the consumption of electricity during the calcination process, the material price of CZ is higher than NZ. However, CZ has a better performance for reducing autogenous shrinkage than NZ. Using CZ can lower the content of chemical admixtures, such as expansive admixture and shrinkage-reducing admixture. In general, CZ shows a better overall performance than NZ.

#### 3.2.2. Development of Internal Relative Humidity (IRH) and Internal Temperature (IT) of the AAS Mortars

**Figure 4:**
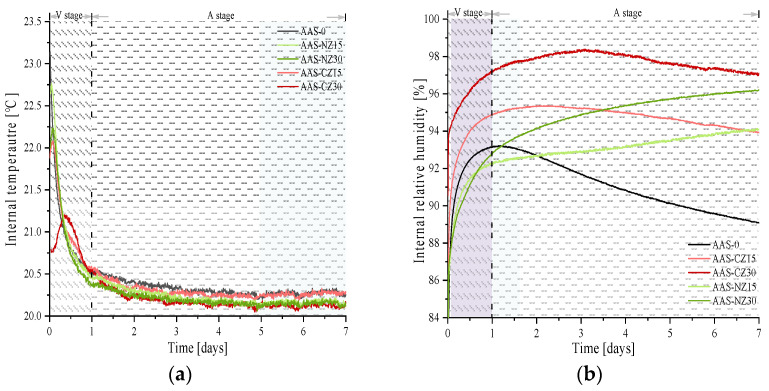
Relationship between the internal temperature (**a**) and internal relative humidity (**b**).

Figure 4 shows the trends in IT (Figure 4a) and IRH (Figure 4b) from zero to seven days of age. First, the IT increased and then decreased to ambient temperature for all groups of the AAS mortars. The order of IT from high to low is as follows: AAS-0 (23.14 °C), AAS-NZ15 (22.76 °C), AAS-NZ30 (22.23 °C), AAS-CZ15 (22.76 °C), and AAS-CZ30 (22.08 °C). Second, according to the IT trend, the reactions of the AAS mortars were divided into two stages: the variable temperature stage (V stage) and the ambient temperature stage (A stage), and the two stages were split at the one-day-old stage.

There were some interesting phenomena that occurred in the changing of IT, as shown in Figure 4b. First, in the V stage, the IRH of all groups of AAS mortars consistently increased. The reasons for this behavior are attributed to the following three factors: (1) the IT of all group specimens was higher than the ambient temperature. The liquid was released and redistributed as condensed liquid overtime in the ink bottle spaces, causing the IRH to increase [37]. (2) As the liquid absorbed by the zeolite sand was continuously released, the IRH of the AAS mortars with internal curing increased [23]. (3) The hydration reaction, however, continuously reduced IRH. Mortars have an increase in IRH because of the combined impact of the three main factors described above. Similar IRH change trends were reported in the study by Fang et al. [38], who found a continuous increase in IRH over a 24 h period for AAS paste.

Second, the IRH curve of AAS-0 began to decrease continuously in the A stage. This behavior was mainly due to the IT approaching ambient temperature. In addition, the decrease in IRH was maintained to a high level due to the continued hydration reaction and the absence of an internal curing liquid within the AAS-0 mortar. In this study, the IRH change trend in the A stage was similar to the results of most other researchers [39]. For example, Song et al. [14] found a continuous decrease in IRH in all AAS mortars without internal curing in which different activators were used.

Third, in the A stage, compared to the AAS mortars with calcined zeolite (the AAS-CZ mortars), the IRH of the AAS mortars with natural zeolite (the AAS-NZ mortars) continuously increased. The continued release of the internal curing liquid from the natural zeolite sand played an important role, resulting in the continued increase of IRH. This behavior is attributed to the different pore structures of the two zeolite sands, as modified zeolite sand can release more of the internal curing liquid at an early age [22].

Finally, the internal curing liquid absorbed by the natural zeolite sand was less than that of the calcined zeolite sand. However, the natural zeolite sand kept the IRH of the mortars high for a longer time. This behavior is mainly related to the pore structure of zeolite sand. Calcined zeolite sand creates more large pores, resulting in some of the internal curing liquid being released in an early age. Moreover, the internal curing fluid absorbed by natural zeolite sand is gradually released at a later time. The different pore structure of zeolite sand resulted in two distinct trends in the internal relative humidity of the AAS mortars.

### 3.3. Isothermal Calorimetry

**Figure 5:**
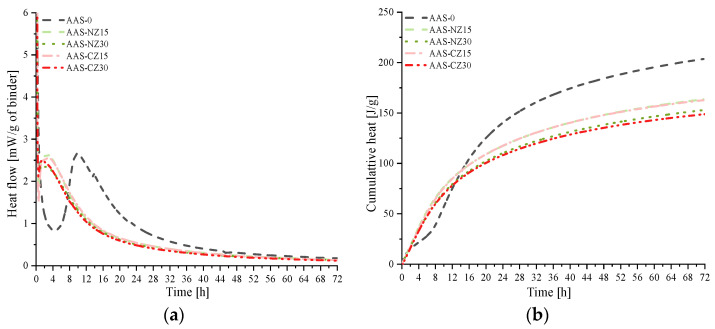
Isothermal calorimetry curves of the AAS mortar specimens for 3 days: (**a**) Heat flow curves; (**b**) cumulative heat curves.

Figure 5 shows the heat flow (Figure 5a) and cumulative heat (Figure 5b) curves of the AAS mortars for curing for 72 h. Zuo et al. [17] previously reported that the heat flow of AAS activated with NaOH as an activator exhibits two main peaks. In this study, because the AAS mortars were mixed outside of glass vials and then put in glass vials, which were placed into the isothermal calorimeter measuring chambers, the first peak of heat flow did not capture completely. The second reaction peak of the AAS mortars with internal curing occurred between 0.5 and 3 h, while the second reaction peak of the AAS mortars without internal curing (AAS-0) occurred between 4.5 and 10 h. Internal curing can shorten the time of the second peak because the Na_2_O content increases [40], which is then released from zeolite sand. The high ionic concentration facilitated the slag reaction. As shown in Figure 5b, from the start to approximately 14 h of mixing, the cumulative heat of the AAS mortars with internal curing was higher than that of the AAS-0 mortars. The reason for this phenomenon is mainly an increase in the ion concentration as a result of the internal curing, which promotes early slag reactions. At three days of age, the final heat of AAS-0, AAS-NZ15, AAS-NZ30, AAS-CZ15, and AAS-CZ30 was 203.6, 163.3, 152.1, 162.2, and 148.2 J/g, respectively. This is because there is an inverse relationship between the concentration of the activator and the heat release [14]. Similar results were reported in a previous study: Gebregziabiher et al. [14] found that high concentrations of NaOH can reduce the heat release of AAS pastes.

### 3.4. Compressive Strength

**Figure 6:**
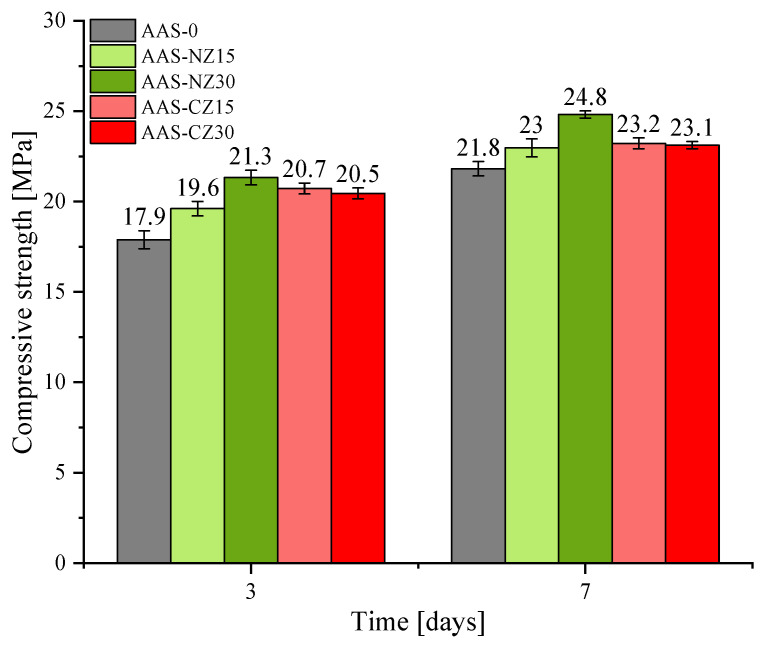
Compressive strength of the alkali-activated slag (AAS) mortars at 3 and 7 days.

The compressive strength of all of the AAS mortars is shown in Figure 6. First, independent of age, compared to AAS-0, the compressive strength increased with the addition of zeolite sand. This behavior was because the addition of zeolite sand released more of the NaOH solution, thereby increasing the ionic concentration. The introduction of ions that can participate in the reaction intensifies the slag reaction, leading to further reaction products, eventually increasing the compressive strength. Second, the compressive strength increased as the substitution of natural zeolite sand increased. This behavior was mainly due to the higher amount of natural zeolite sand increasing the ion concentration, which led to a greater slag reaction and increased hydration products, resulting in increased compressive strength. However, the compressive strength of AAS-CZ15 and AAS-CZ30 was almost similar. Although the AAS-CZ30 mortar provided more ions, an addition of 30% of calcined zeolite introduced additional pores. The involvement of pores has a more direct effect on the strength, so a higher content of calcined zeolite did not significantly increase the compressive strength, but instead exhibited a trend similar to the compressive strength of AAS-CZ15. In addition, the justification for the use of NZ or CZ is dependent on the purpose of utilizations. When the main purpose is reducing autogenous shrinkage, CZ is a better choice than NZ because CZ can make lower autogenous shrinkage than NZ. On the other hand, when the main purpose is high early-age strength, NZ is a better choice because concrete containing NZ shows a slightly higher early-age strength than CZ.

### 3.5. Ultrasonic Characterization

**Figure 7:**
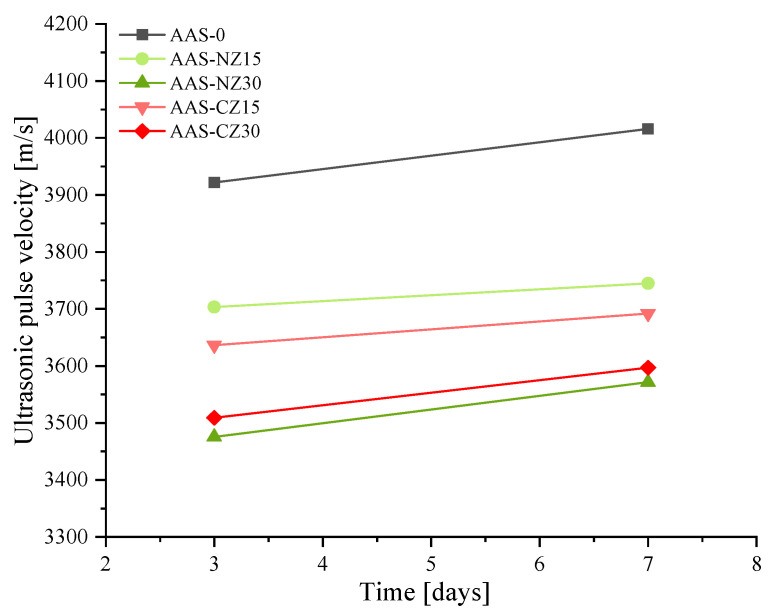
Ultrasonic pulse velocity values of the alkali-activated slag (AAS) mortars at 3 and 7 days.

Figure 7 shows the ultrasonic pulse velocity of the AAS mortars at three and seven days. The ultrasonic pulse velocity of the AAS mortars varied with the pore structure. In previous studies, it was reported that ultrasonic pulse velocity values increase as the compressive strength also increases [41]. However, the results in this study seem to differ to the above conclusion. First, the ultrasonic pulse velocity values increased with curing age. This phenomenon is mainly attributed to the further hydration of the mortars at seven days of age, which formed more hydration products, resulting in a denser structure and an increase in ultrasonic pulse velocity. Second, regardless of the types of zeolite added, as the replacement amount of zeolite sand increased, the ultrasonic pulse velocity measurements decreased. At seven days of age, when 30 wt.% of zeolite sand was used, lower values of 3400–3500 m/s were observed. This behavior was mainly due to the additional pores added by the 30 wt.% of zeolite sand, resulting in more pores in the AAS mortars and lower ultrasonic pulse velocity values. Finally, at seven days, the ultrasonic pulse velocity of AAS-0, AAS-NZ15, AAS-NZ30, AAS-CZ15, and AAS-CZ30 was 4016, 3745, 3571.5, 3692, and 3597 m/s, respectively. The ultrasonic pulse velocity values of the AAS mortars with the same replacement rate of zeolite sand were similar. This behavior was mainly due to the fact that for the same type of zeolite sand, high replacement meant that the additional pores added caused a decrease in the ultrasonic pulse velocity values. In summary, the formation of hydration products can enhance the ultrasonic pulse velocity. However, the addition of zeolite sand introduces additional pores to reduce the ultrasonic pulse velocity. The final results of the ultrasonic pulse velocity depend on the competing results of the above factors.

### 3.6. Surface Electrical Resistivity (SER)

**Figure 8:**
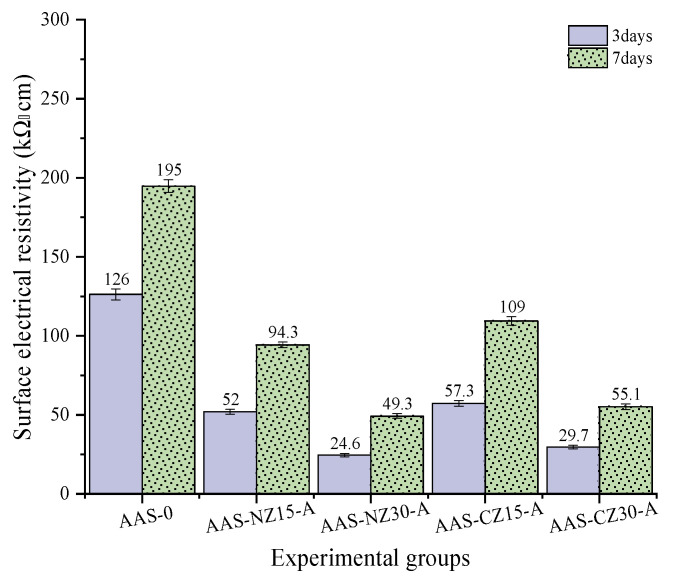
Surface electrical resistivity of the alkali-activated slag (AAS) mortars at 3 and 7 days.

Surface electrical resistivity testing of concrete can be used to evaluate the resistivity of water-saturated concrete for a quick indication of the resistance of said concrete to chloride ion penetration. Since the resistivity of concrete specimens can be measured at different ages, the resistivity over time can be used to assess the microstructure development rate of the concrete due to hydration and secondary reactions. The resistivity of concrete is influenced by multiple factors, such as pore size distribution, pore interconnections, pore fluid conductivity, saturation, and temperature variation [42]. Surface electrical resistivity testing can be used as an indirect measure of porosity and diffusivity. The electrical current through concrete is generated by an electrolysis process, which is caused by the flow of ions, such as Na^+^, K^+^, Ca^2+^, SO_4_^2+^, and OH^−^, present in the pore solution. The concentration of OH^−^ in the pore solution depends on the alkali concentration [43] and, as per this study, on the presence of internal curing agents and the effect of internal curing. Figure 8 shows the surface electrical resistivity results for the AAS mortar at three and seven days of age. Regardless of the curing age, the surface electrical resistivity value decreased significantly with the addition of zeolite sand. This phenomenon is mainly attributed to the internal curing agent providing an internal curing liquid to the pore network, leading to an increase in the alkali concentration, thereby resulting in an increase in the ionic conductivity and a decrease in the surface electrical resistivity. In addition, the surface electrical resistivity of the mortars was significantly lower for a 30 wt.% replacement compared to the AAS mortars containing 15 wt.% of zeolites. This is mainly attributed to the fact that the higher the zeolite content, the more internal curing fluid absorbed, and as the internal curing fluid is released, the alkali concentration increases, thus increasing the ionic conductivity, resulting in a reduction in the surface electrical resistivity.

## 4. Conclusions

This study shows experimental investigations of heat flow-strength-autogenous shrinkage-durability of alkali-activated slag (AAS) mortars. Calcined zeolite and natural zeolite sand were used to replace natural sand at 15 wt.% and 30 wt.%. Various properties of the AAS mortar were then measured: setting time, autogenous shrinkage, compressive strength, ultrasonic pulse velocity, and surface electrical resistivity. The following conclusions were obtained:

1. Compared to AAS-0 and AAS-NZ, the addition of calcined zeolite sand can extend the setting time (72 min more) of AAS mortars and make it possible to alleviate the problem of AAS mortars being challenging to process in cast-in-place concrete due to rapid setting.
2. When the amount of calcined zeolite added was up to 30%, the autogenous shrinkage of the AAS mortars at seven days of age was almost offset by approximately −11.65 μm/m. Compared to the autogenous shrinkage of the control group (approximately −324.68 μm/m), the 30% replacement amount of calcined zeolite reduced the autogenous shrinkage by 96.4%.
3. In this study, the autogenous shrinkage development of the AAS mortars was divided into two stages, i.e., variable temperature stage and ambient temperature stage. The time division point between the two stages was at one day of age. For the internal relative humidity, in the variable temperature stage, all of the AAS mortars showed an increase. In the ambient temperature stage, AAS-0 decreased due to the absence of a continuously available internal curing liquid. However, different internal relative humidity trends were observed for the AAS mortars with internal curing. Among them, AAS-CZ exhibited an increasing and then decreasing behavior, while AAS-NZ exhibited a continuous increase.
4. Compared to AAS-0, the second peak of the AAS mortars with internal curing appeared significantly earlier by approximately 7 h, due to the increased concentration of Na_2_O from the release of the internal curing liquid. The total heat of the hydration release decreased as the NaOH concentration increased.
5. Compared to the control group, the compressive strength of the AAS mortars with internal curing increased at both three and seven days of age. Comparing the strength of the AAS mortars with different zeolite sands, it was found that although the high content of internal curing liquid had a positive effect on the compressive strength, the introduction of calcined zeolite itself increased the additional porosity of the AAS matrix. Therefore, natural zeolite at a substitution rate of 30% results in AAS mortars exhibiting the best compressive strength.
6. The formation of hydration products can enhance the ultrasonic pulse velocity. On the contrary, the addition of zeolite introduces additional pores to reduce the ultrasonic pulse velocity. The final results of the ultrasonic pulse velocity depend on the competing results of these two factors. In addition, the introduction of internal curing decreases the surface electrical resistivity due to the continuous supply of internal curing liquid, thereby increasing the ionic conductivity.

## Figures and Tables

**Table 1:** Chemical compositions of the slag and zeolite sand.

Materials	SiO_2_	CaO	Al_2_O_3_	MgO	Fe_2_O_3_	SO_3_	K_2_O	TiO_2_	MnO	Loss
GGBFS (%)	32.25	38.85	15.78	7.13	0.56	2.68	0.65	0.44	0.17	1.25
Zeolite sand (%)	65.39	1.91	13.43	1.36	1.63	−	−	−	−	16.27

GGBFS, granulated ground blast furnace slag.

**Table 2:** Mix proportions based on mass and volume.

Mix	Slag	Sand/Binder	Liquid/Binder			Aggregate (%)			Mixture Based on Volume (kg/m^3^)				
			Basic Liquid/Binder	Additional Liquid/Binder	Total Liquid/Binder	Sand	Natural Zeolite	Calcined Zeolite	Slag	Alkali Solution	Standard Sand	Natural Zeolite	Calcined Zeolite
AAS-0	1	2.0	0.55	0	0.55	100	−	−	613.84	337.61	1227.68	−	−
AAS-NZ15	1	2.0	0.55	0.017	0.567	85	15	−	601.00	340.77	1021.70	180.30	−
AAS-NZ30	1	2.0	0.55	0.034	0.584	70	30	−	588.69	343.80	824.17	353.22	−
AAS-CZ15	1	2.0	0.55	0.071	0.621	85	−	15	575.77	357.56	978.81	−	172.73
AAS-CZ30	1	2.0	0.55	0.142	0.692	70	−	30	542.15	375.17	759.01	−	325.29

## Data Availability

The data presented in this study are available from the corresponding author upon a reasonable request.
